# An open science resource for establishing reliability and reproducibility in functional connectomics

**DOI:** 10.1038/sdata.2014.49

**Published:** 2014-12-09

**Authors:** Xi-Nian Zuo, Jeffrey S Anderson, Pierre Bellec, Rasmus M Birn, Bharat B Biswal, Janusch Blautzik, John C.S Breitner, Randy L Buckner, Vince D Calhoun, F. Xavier Castellanos, Antao Chen, Bing Chen, Jiangtao Chen, Xu Chen, Stanley J Colcombe, William Courtney, R Cameron Craddock, Adriana Di Martino, Hao-Ming Dong, Xiaolan Fu, Qiyong Gong, Krzysztof J Gorgolewski, Ying Han, Ye He, Yong He, Erica Ho, Avram Holmes, Xiao-Hui Hou, Jeremy Huckins, Tianzi Jiang, Yi Jiang, William Kelley, Clare Kelly, Margaret King, Stephen M LaConte, Janet E Lainhart, Xu Lei, Hui-Jie Li, Kaiming Li, Kuncheng Li, Qixiang Lin, Dongqiang Liu, Jia Liu, Xun Liu, Yijun Liu, Guangming Lu, Jie Lu, Beatriz Luna, Jing Luo, Daniel Lurie, Ying Mao, Daniel S Margulies, Andrew R Mayer, Thomas Meindl, Mary E Meyerand, Weizhi Nan, Jared A Nielsen, David O’Connor, David Paulsen, Vivek Prabhakaran, Zhigang Qi, Jiang Qiu, Chunhong Shao, Zarrar Shehzad, Weijun Tang, Arno Villringer, Huiling Wang, Kai Wang, Dongtao Wei, Gao-Xia Wei, Xu-Chu Weng, Xuehai Wu, Ting Xu, Ning Yang, Zhi Yang, Yu-Feng Zang, Lei Zhang, Qinglin Zhang, Zhe Zhang, Zhiqiang Zhang, Ke Zhao, Zonglei Zhen, Yuan Zhou, Xing-Ting Zhu, Michael P Milham

**Affiliations:** 1 Key Laboratory of Behavioral Science and Magnetic Resonance Imaging Research Center, Institute of Psychology, Chinese Academy of Sciences, Chaoyang, Beijing 100101, China; 2 Faculty of Psychology, Southwest University, Beibei, Chongqing 400715, China; 3 Division of Neuroradiology, University of Utah, Salt Lake City, Utah 84132, USA; 4 Unité de neuroimagerie fonctionnelle, Centre de recherche de l’institut universitaire de gériatrie de Montréal, Université de Montréal, Montreal, Quebec, Canada H3W 1W5; 5 Department of Psychiatry, University of Wisconsin—Madison, Madison, Wisconsin 53719, USA; 6 Department of Biomedical Engineering, New Jersey Institute of Technology, Newark, New Jersey 07102, USA; 7 Institute of Clinical Radiology, Ludwig-Maximilians-University, 80336 Munich, Germany; 8 Centre for Studies on Prevention of Alzheimer’s Disease, Department of Psychiatry, Douglas Institute, McGill University Faculty of Medicine, Montreal, Quebec, Canada H4H 1R3; 9 Department of Psychology, Harvard University, Cambridge, Massachussetts 02138, USA; 10 Mind Research Network, Albuquerque, New Mexico 87106, USA; 11 Nathan S. Kline Institute for Psychiatric Research, Orangeburg, New York 10962, USA; 12 Phyllis Green and Randolph Cōwen Institute for Pediatric Neuroscience, the Child Study Center at NYU Langone Medical Center, New York, New York 10016, USA; 13 Center for Cognition and Brain Disorders, Hangzhou Normal University, Gongshu, Hangzhou, Zhejiang 311121, China; 14 Center for the Developing Brain, Child Mind Institute, New York, New York 10022, USA; 15 University of Chinese Academy of Sciences, Shijingshan, Beijing 100049, China; 16 State Key Laboratory of Brain and Cognitive Science, Institute of Psychology, Chinese Academy of Sciences, Chaoyang, Beijing 100101, China; 17 Huaxi MR Research Center (HMRRC), Department of Radiology, West China Hospital of Sichuan University, Wuhou, Chengdu, Sichuan 610041, China; 18 Max Planck Research Group for Neuroanatomy & Connectivity, Max Planck Institute for Human Cognitive and Brain Sciences, 04103 Leipzig, Germany; 19 Department of Neurology, Xuanwu Hospital, Capital Medical University, Xicheng, Beijing 100053, China; 20 State Key Laboratory of Cognitive Neuroscience and Learning and IDG/McGovern Institute for Brain Research, Beijing Normal University, Haidian, Beijing 100875, China; 21 Department of Psychology, Yale University, New Haven, Connecticut 06511, USA; 22 Department of Psychological and Brain Sciences, Center for Cognitive Neuroscience, Dartmouth College, Hanover, New Hampshire 03755, USA; 23 Brainnetome Center and National Laboratory of Pattern Recognition, Institute of Automation, Chinese Academy of Sciences, Haidian, Beijing 100190, China; 24 Virginia Tech Carilion Research Institute, Roanoke, Virginia 24016, USA; 25 Department of Radiology, Xuanwu Hospital, Capital Medical University, Xicheng, Beijing 100053, China; 26 Department of Medical Imaging, Jinling Hospital, School of Medicine, Nanjing University, Xuanwu, Nanjing, Jiangsu 210002, China; 27 Department of Psychiatry, School of Medicine, University of Pittsburgh, Pittsburgh, Pennsylvania 15213, USA; 28 Beijing Key Laboratory of Learning and Cognition, Department of Psychology, Capital Normal University, Haidian, Beijing 100048, China; 29 Department of Neurosurgery, Huashan Hospital, Fudan University, Jingan, Shanghai 200040, China; 30 Department of Biomedical Engineering, University of Wisconsin—Madison, Madison, Wisconsin 53705, USA; 31 Department of Radiology, University of Wisconsin—Madison, Madison, Wisconsin 53705, USA; 32 Department of Psychiatry, Huashan Hospital, Fudan University, Jingan, Shanghai 200021, China; 33 Department of Radiology, Huashan Hospital, Fudan University, Jingan, Shanghai 200040, China; 34 Department of Neurology, Max Planck Institute for Human Cognitive and Brain Sciences, Leipzig 04103, Germany; 35 Department of Psychiatry, Renmin Hospital of Wuhan University, Wuhan, Hubei 430060, China

## Abstract

Efforts to identify meaningful functional imaging-based biomarkers are limited by the ability to reliably characterize inter-individual differences in human brain function. Although a growing number of connectomics-based measures are reported to have moderate to high test-retest reliability, the variability in data acquisition, experimental designs, and analytic methods precludes the ability to generalize results. The Consortium for Reliability and Reproducibility (CoRR) is working to address this challenge and establish test-retest reliability as a minimum standard for methods development in functional connectomics. Specifically, CoRR has aggregated 1,629 typical individuals’ resting state fMRI (rfMRI) data (5,093 rfMRI scans) from 18 international sites, and is openly sharing them via the International Data-sharing Neuroimaging Initiative (INDI). To allow researchers to generate various estimates of reliability and reproducibility, a variety of data acquisition procedures and experimental designs are included. Similarly, to enable users to assess the impact of commonly encountered artifacts (for example, motion) on characterizations of inter-individual variation, datasets of varying quality are included.

## Background & Summary

Functional connectomics is a rapidly expanding area of human brain mapping^1–4^. Focused on the study of functional interactions among nodes in brain networks, functional connectomics is emerging as a mainstream tool to delineate variations in brain architecture among both individuals and populations^5–8^. Findings that established network features and well-known patterns of brain activity elicited via task performance are recapitulated in spontaneous brain activity patterns captured by resting-state fMRI (rfMRI)^3–6,9–12^, have been critical to the wide-spread acceptance of functional connectomics applications.

A growing literature has highlighted the possibility that functional network properties may explain individual differences in behavior and cognition^4,7,8^—the potential utility of which is supported by studies that suggest reliability for commonly used rfMRI measures^13^. Unfortunately, the field lacks a data platform by which researchers can rigorously explore the reliability of the many indices that continue to emerge. Such a platform is crucial for the refinement and evaluation of novel methods, as well as those that have gained widespread usage without sufficient consideration of reliability. Equally important is the notion that quantifying the reliability and reproducibility of the myriad connectomics-based measures can inform expectations regarding the potential of such approaches for biomarker identification^13–16^.

To address these challenges, the Consortium for Reliability and Reproducibility (CoRR) has aggregated previously collected test-retest imaging datasets from more than 36 laboratories around the world and shared them via the 1000 Functional Connectomes Project (FCP)^5,17^ and its International Neuroimaging Data-sharing Initiative (INDI)^18^. Although primarily focused on rfMRI, this initiative has worked to promote the sharing of diffusion imaging data as well. It is our hope that among its many possible uses, the CoRR repository will facilitate the: (1) Establishment of test-retest reliability and reproducibility for commonly used MR-based connectome metrics, (2) Determination of the range of variation in the reliability and reproducibility of these metrics across imaging sites and retest study designs, (3) Creation of a standard/benchmark test-retest dataset for the evaluation of novel metrics.

Here, we provide an overview of all the datasets currently aggregated by CoRR, and describe the standardized metadata and technical validation associated with these datasets, thereby facilitating immediate access to these data by the wider scientific community. Additional datasets, and richer descriptions of some of the studies producing these datasets, will be published separately (for example, A high resolution 7-Tesla rfMRI test-retest dataset with cognitive and physiological measures^19^). A list of all papers describing these individual studies will be maintained and periodically updated at the CoRR website (http://fcon_1000.projects.nitrc.org/indi/CoRR/html/data_citation.html).

## Methods

### Experimental design

At the time of submission, CoRR has received 40 distinct test-retest datasets that were independently collected by 36 imaging groups at 18 institutions. All CoRR contributions were based on studies approved by a local ethics committee; each contributor’s respective ethics committee approved submission of de-identified data. Data were fully deidentified by removing all 18 HIPAA (Health Insurance Portability and Accountability)-protected health information identifiers, and face information from structural images prior to contribution. All data distributed were visually inspected before release. While all samples include at least one baseline scan and one retest scan, the specific designs and target populations employed across samples vary given the aggregation strategy used to build the resource. Since many individual (uniformly collected) datasets have reasonably large sample sizes allowing stable test-retest estimates, this variability across datasets provides an opportunity to generalize reliability estimates across scanning platforms, acquisition approaches, and target populations. The range of designs included is captured by the following classifications:

- ***Within-Session Repeat.***
- o *Scan repeated on same day*
- o *Behavioral condition may or may not vary across scans depending on sample*
- ***Between-Session Repeat.***
- o *Scan repeated one or more days later*
- o *In most cases less than one week*
- ***Between-Session Repeat (Serial).***
- o *Scan is repeated for 3 or more sessions in a short time-frame that is believed to be developmentally stable*
- ***Between-Session Repeat (Longitudinal developmental).***
- o *Scan repeated at a distant time-point not believed to be developmentally equivalent. There is no exact definition of the minimum time for detecting developmental effects across scans, though designs typically span at least 3–6 months*
- ***Hybrid Design.***
- o *Scans repeated one or more times on same day, as well as across one or more sessions*

Table 1 presents an overview of the specific samples included in CoRR (Data Citations 1, 2, 3, 4, 5, 6, 7, 8, 9, 10, 11, 12, 13, 14, 15, 16, 17, 18, 19, , 21, 22, 23, 24, 25, 26, 27, 28, 29, 30, 31). The vast majority included a single retest scan (48% within-session, 52% between-session). Three samples employed serial scanning designs, and one sample had a longitudinal developmental component. Most samples included presumed neurotypical adults; exceptions include the pediatric samples from Institute of Psychology at Chinese Academy of Sciences (IPCAS 2/7), University of Pittsburgh School of Medicine (UPSM) and New York University (NYU) and the lifespan samples from Nathan Kline Institute (NKI 1).

## Data Records

### Data privacy

Prior to contribution, each investigator confirmed that the data in their contribution was collected with the approval of their local ethical committee or institutional review board, and that sharing via CoRR was in accord with their policies. In accord with prior FCP/INDI policies, face information was removed from anatomical images (FullAnonymize.sh V1.0b; http://www.nitrc.org/frs/shownotes.php?release_id=1902) and Neuroimaging Informatics Technology Initiative (NIFTI) headers replaced prior to open sharing to minimize the risk of re-identification.

### Distribution for use

CoRR data sets can be accessed through either the COllaborative Informatics and Neuroimaging Suite (COINS) Data Exchange (http://coins.mrn.org/dx)^20^, or the Neuroimaging Informatics Tools and Resources Clearinghouse (NITRC; http://fcon_1000.projects.nitrc.org/indi/CoRR/html/index.html). CoRR datasets at the NITRC site are stored in .tar files sorted by site, each containing the necessary imaging data and phenotypic information. The COINS Data Exchange offers an enhanced graphical query tool, which enables users to target and download files in accord with specific search criteria. For each sharing venue, a user login must be established prior to downloading files. There are several groups of samples which were not included in the data analysis as they were in the data contribution/upload, preparation or correction stage at the time of analysis: Intrinsic Brain Activity, Test-Retest Dataset (IBATRT), Dartmouth College (DC 1), IPCAS 4, Hangzhou Normal University (HNU 2), Fudan University (FU 1), FU 2, Chengdu Huaxi Hospital (CHH 1), Max Planck Institute (MPG 1)^19^, Brain Genomics Superstruct Project (GSP) and New Jersey Institute of Technology (NJIT 1) (see more details on these sites at the CoRR website). Table 1 provides a static representation of the samples included in CoRR at the time of submission.

### Imaging data

Consistent with its popularity in the imaging community and prior usage in FCP/INDI efforts, the NIFTI file format was selected for storage of CoRR imaging datasets, independent of modalities such as rfMRI, structural MRI (sMRI) and dMRI. Tables 2, 3, 4 (available online only) provide descriptions of the MRI sequences used for the various modalities for each of the imaging data file types.

### Phenotypic information

All phenotypic data are stored in comma separated value (.csv) files. Basic information such as age and gender has been collected for each site to facilitate aggregation with minimal demographic variables. Table 5 (available online only) depicts the data legend provided to CoRR contributors.

## Technical Validation

**Figure 1:**
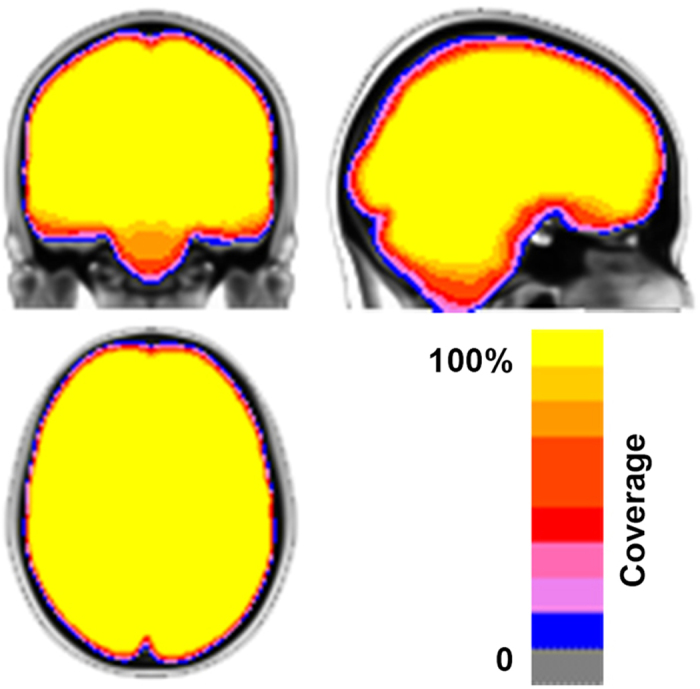
Summary map of brain coverage for rfMRI scans in CoRR (*N*=5,093). The color indicates the coverage ratio of rfMRI scans.

Consistent with the established FCP/INDI policy, all data contributed to CoRR was made available to users regardless of data quality. Justifications for this decision include the lack of consensus within the functional imaging community on criteria for quality assurance, and the utility of ‘lower quality’ datasets for facilitating the development of artifact correction techniques. For CoRR, the inclusion of datasets with significant artifacts related to factors such as motion are particularly valuable, as it enables the determination of the impact of such real-world confounds on reliability and reproducibility^21,22^. However, the absence of screening for data quality in the data release does not mean that the inclusion of poor quality datasets in imaging analyses is routine practice for the contributing sites. Figure 1 provides a summary map describing the anatomical coverage for rfMRI scans included in the CoRR dataset.

To facilitate quality assessment of the contributed samples and selection of datasets for analyses by individual users^23^, we made use of the Preprocessed Connectome Project quality assurance protocol (http://preprocessed-connectomes-project.github.io), which includes a broad range of quantitative metrics commonly used in the imaging literature for assessing data quality, as follows. They are itemized below:

- ***Spatial Metrics (sMRI, rfMRI)***
- o *Signal-to-Noise Ratio (SNR)* ^24^. The mean within gray matter values divided by the standard deviation of the air values.
- o *Foreground to Background Energy Ratio (FBER)*
- o *Entropy Focus Criteria (EFC)* ^25^. Shannon’s entropy is used to summarize the principal directions distribution.
- o *Smoothness of Voxels* ^26^. The full-width half maximum (FWHM) of the spatial distribution of image intensity values.
- o *Ghost to Signal Ratio (GSR) (only rfMRI)* ^27^. A measure of the mean signal in the ‘ghost’ image (signal present outside the brain due to acquisition in the phase encoding direction) relative to mean signal within the brain.
- o *Artifact Detection (only sMRI)* ^28^. The proportion of voxels with intensity corrupted by artifacts normalized by the number of voxels in the background.
- o *Contrast-to-Noise Ratio (CNR) (only sMRI)* ^24^. Calculated as the mean of the gray matter values minus the mean of the white matter values, divided by the standard deviation of the air values.
- ***Temporal Metrics (rfMRI)***
- o Head Motion
- ▪*Mean framewise displacement (FD)* ^29^. A measure of subject head motion, which compares the motion between the current and previous volumes. This is calculated by summing the absolute value of displacement changes in the x, y and z directions and rotational changes about those three axes. The rotational changes are given distance values based on the changes across the surface of a 50 mm radius sphere.
- ▪*Percent of volumes with FD greater than 0.2 mm*
- ▪*Standardized DVARS.* The spatial standard deviation of the temporal derivative of the data (D referring to temporal derivative of time series, VARS referring to root-mean-square variance over voxels)^29^, normalized by the temporal standard deviation and temporal autocorrelation (http://blogs.warwick.ac.uk/nichols/entry/standardizing_dvars).
- o *General*
- ▪*Outlier Detection.* The mean fraction of outliers found in each volume using 3dTout command in the software package for Analysis of Functional NeuroImages (AFNI: http://afni.nimh.nih.gov/afni).
- ▪*Median Distance Index.* The mean distance (1-spearman’s rho) between each time-point’s volume and the median volume using AFNI’s 3dTqual command.
- ▪*Global Correlation (GCOR)* ^30^. The average of the entire brain correlation matrix, which is computed as the brain-wide average time series correlation over all possible combinations of voxels.

**Figure 2:**
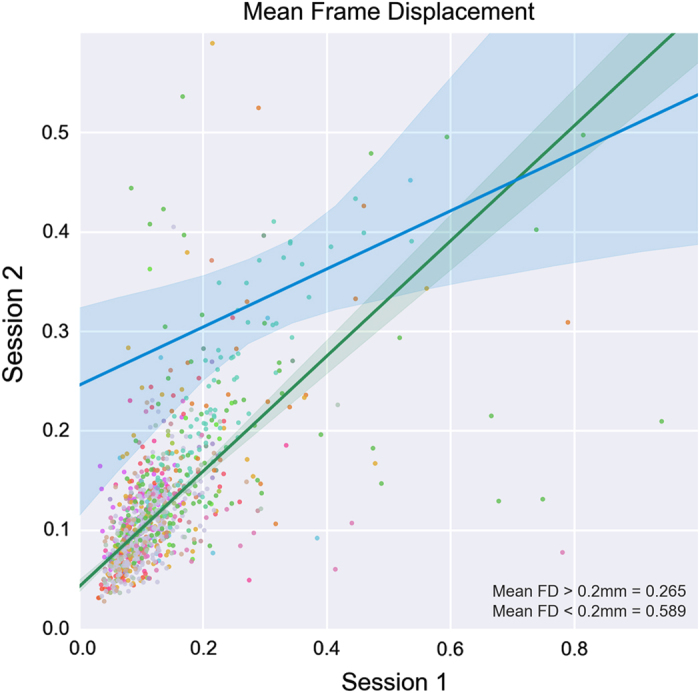
Test-retest plots of in-scanner head motion during rfMRI. Total 1019 subjects who have at least two rfMRI sessions are selected. The green line indicates the correlation between the two sessions within the lower motion datasets (mean FD<0.2 mm). The blue line indicates the correlation for the higher motion datasets (mean FD >0.2 mm).

Imaging data preprocessing was carried out with the Configurable Pipeline for the Analysis of Connectomes (C-PAC: http://www.nitrc.org/projects/cpac). Results for the sMRI images (spatial metrics) are depicted in Supplementary Figure 1, for the rfMRI scans in Supplementary Figure 2 (general spatial and temporal metrics) and Supplementary Figure 3 (head motion). For both sMRI and rfMRI, the battery of quality metrics revealed notable variations in image properties across sites. It is our hope that users will explore the impact of such variations in quality on the reliability of data derivatives, as well as potential relationships with acquisition parameters. Recent work examining the impact of head motion on reliability suggests the merits of such lines of questioning. Specifically, Yan and colleagues found that motion itself has moderate test-retest reliability, and appears to contribute to reliability when low, though it compromises reliability when high^31–33^. Although a comprehensive examination of this issue is beyond the scope of the present work, we did verify that motion does have moderate test-retest reliability in the CoRR datasets (see Figure 2) as previously suggested. Interestingly, this relationship appeared to be driven by the lower motion datasets (mean FD<0.2mm). Future work will undoubtedly benefit from further exploration of this phenomena and its impact of findings.

Beyond the above quality control metrics, a minimal set of rfMRI derivatives for the datasets were calculated for the datasets included in CoRR to further facilitate comparison of images across sites:

- o *Fractional Amplitude of Low Frequency Fluctuations (fALFF)* ^34,35^. The total power in the low frequency range (0.01–0.1 Hz) of an fMRI image, normalized by the total power across all frequencies measured in that same image.
- o *Voxel-Mirrored Homotopic Connectivity (VMHC)* ^36,37^. The functional connectivity between a pair of geometrically symmetric, inter-hemispheric voxels.
- o *Regional Homogeneity (ReHo)* ^38–40^. The synchronicity of a voxel’s time series and that of its nearest neighbors based on Kendall’s coefficient of concordance to measure the local brain functional homogeneity.
- o *Intrinsic Functional Connectivity (iFC) of Posterior Cingulate Cortex (PCC)* ^41^. Using the mean time series from a spherical region of interest (diameter=8 mm) centered in PCC (x=−8, y=−56, z=26)^42^, functional connectivity with PCC is calculated for each voxel in the brain using Pearson’s correlation (results are Fisher r-to-z transformed).

**Figure 3:**
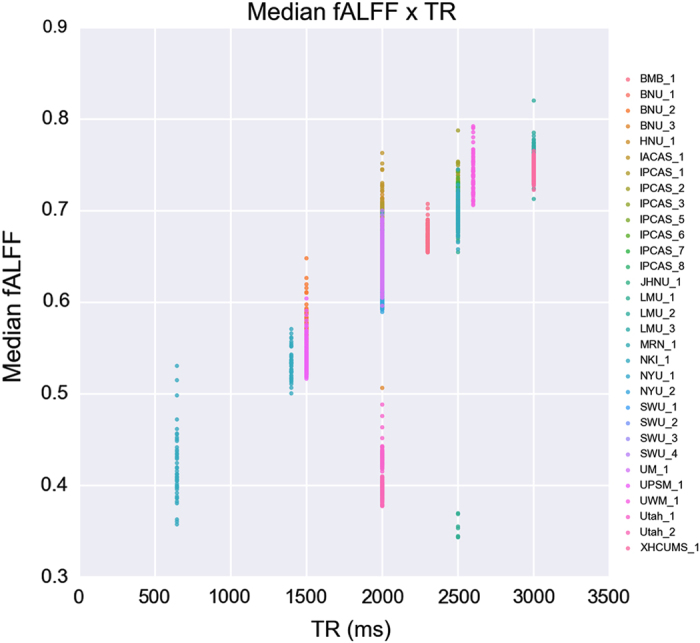
Individual differences in fALFF and the temporal sampling rate (TR). Median fALFF values across each individual whole brains are plotted against the corresponding TR for each site. Different colors indicate labels of different sites.

To enable rapid comparison of derivatives, we: (1) calculated the 50th, 75th, and 90th percentile scores for each participant, and then (2) calculated site means and standard deviations for each of these scores (see Table 6 (available online only)). We opted to not use increasingly popular standardization approaches (for example, mean-regression, mean centering +/− variance normalization) in the calculation of derivative values, as the test-retest framework provides users a unique opportunity to consider the reliability of site-related differences. As can be seen in Supplementary Figure 4, for all the derivatives, the mean value or coefficient of variation obtained for a site was highly reliable. In the case of fALFF, site-specific differences can be directly related to the temporal sampling rate (that is, TR; see Figure 3), as lower TR datasets include a broader range of frequencies in the denominator—thereby reducing the resulting fALFF scores (differences in aliasing are likely to be present as well). This note of caution about fALFF raises the general issue that rfMRI estimates can be highly sensitive to acquisition parameters^7,13^. Specific factors contributing to differences in the other derivatives are less obvious (it is important to note that the correlation-based derivatives have some degree of standardization inherent to them). Interestingly, the coefficient of variation across participants also proved to be highly reliable for the various derivatives; while this may point to site-related differences in the ability to detect differences across participants, it may also be some reflection of the specific populations obtained at a site (or the sample size). Overall, these site-related differences highlight the potential value of *post-hoc* statistical standardization approaches, which can be used to handle unaccounted for sources of variation within-site as well^43^.

**Figure 4:**
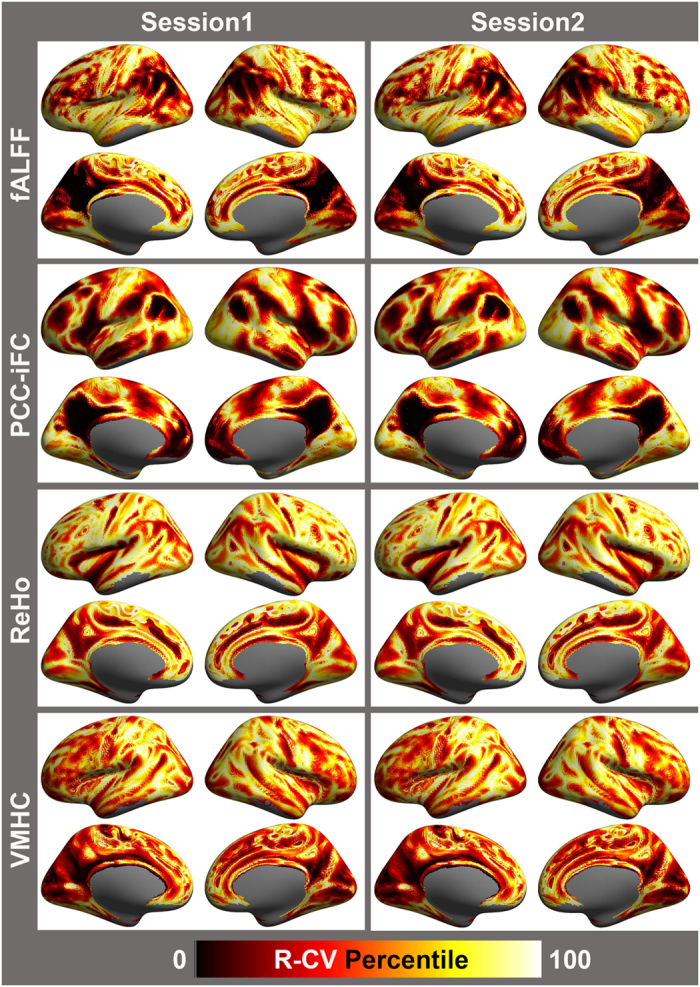
Test-retest plots of individual variation-related functional boundaries. Detection of functional boundaries was achieved via examination of voxel-wise coefficients of variation (CV) for fALFF, PCC, ReHo and VMHC maps. For the purpose of visualization, coefficients of variation were rank-ordered, whereby the relative degree of variation across participants at a given voxel, rather than the actual value, was plotted to better contrast brain regions. Ranking coefficients of variation (R-CV) efficiently identified regions of greatest inter-individual variability, thus delineating putative functional boundaries.

Finally, in Figure 4, we demonstrate the ability of the CoRR datasets to: (1) replicate prior work showing regional differences in inter-individual variation for the various derivatives that occur at ‘transition zones’ or boundaries between functional areas (even after mean-centering and variance normalization), and (2) show them to be highly reproducible across imaging sessions in the same sample. It is our hope that this demonstration will spark future work examining inter-individual variation in these boundaries and their functional relevance. These surface renderings and visualizations are carried out with the Connectome Computation System (CCS) documented at http://lfcd.psych.ac.cn/ccs.html and will be released to the public via github soon (https://github.com/zuoxinian/CCS).

To facilitate replication of our work, for each of Figures 1, 2,3 and Supplementary Figures 1–4, we include a variable in the COINS phenotypic data that indicates whether or not each dataset was included in the analyses depicted. We also included this information in the phenotypic files on NITRC.

## Usage Notes

While formal test-retest reliability or reproducibility analyses are beyond the scope of the present data description, we illustrate the broad range of potential questions that can be answered for rfMRI, dMRI and sMRI using the resource. These include the impact of:

- Acquisition parameters^7,38,44^
- Image quality^13^
- Head motion^7,30,38,43,45^
- Image processing decisions^13,30,38,43,46–48^ (for example, nuisance signal regression for rfMRI, spatial normalization algorithms, computational space)
- Standardization approaches^43^
- *Post-hoc* analytic choices^13,49,50^
- Age^51–53^

Of note, at present, the vast majority of studies do not collect physiological data, and this is reflected in the CoRR initiative. With that said, recent advances in model-free correction (for example, ICA-FIX^54,55^, CORSICA^56^, PESTICA^57^, PHYCAA^58,59^) can be of particular value in the absence of physiological data.

Additional questions may include:

- How reliable are image quality metrics?
- How does reliability and reproducibility impact prediction accuracy?
- How do imaging modalities (for example, rfMRI, dMRI, sMRI) differ with respect to reproducibility and reliability? And within modality, are some derivatives more reliable than others?
- Can reliability and reproducibility be used to optimize imaging analyses? How can such optimizations avoid being driven by artifacts such as motion?
- How much information regarding inter-individual variation is shared and distinct among imaging metrics?
- Which features best differentiate one individual from another?

One example analytic framework that can be used with the CoRR test-retest datasets is Non-Parametric Activation and Influence Reproducibility reSampling (NPAIRS^60^). By combining prediction accuracy and reproducibility, this computational framework can be used to assess the relative merits of differing image modalities, image metrics, or processing pipelines, as well as the impact of artifacts^61–63^.

Open access connectivity analysis packages that may be useful (list adapted from http://RFMRI.org):

- Brain Connectivity Toolbox (BCT; MATLAB)^64^
- BrainNet Viewer (BNV; MATLAB)^65^
- Configurable Pipeline for the Analysis of Connectomes (C-PAC; PYTHON)^66^
- CONN: functional connectivity toolbox (CONN; MATLAB)^67^
- Connectome Computation System (CCS; SHELL/MATLAB)^13,38,39^
- Dynamic Causal Model (DCM; MATLAB) as part of Statistical Parameter Mapping (SPM)^68,69^
- Data Processing Assistant for Resting-State FMRI (DPARSF; MATLAB)^70^
- Functional and Tractographic Connectivity Analysis Toolbox (FATCAT; C) as part of AFNI^71,72^
- Seed-based Functional Connectivity (FSFC; SHELL) as part of FreeSurfer^73^
- Graph Theory Toolkit for Network Analysis (GRETNA; MATLAB)^74^
- Group ICA of FMRI Toolbox (GIFT; MATLAB)^75^
- Multivariate Exploratory Linear Optimized Decomposition into Independent Components (MELODIC; C) as part of FMRIB Software Library (FSL)^76,77^
- Neuroimaging Analysis Kit (NIAK: MATLAB/OCTAVE)^78^
- Ranking and averaging independent component analysis by reproducibility (RAICAR; MATLAB)^79,80^
- Resting-State fMRI Data Analysis Toolkit (REST; MATLAB)^81^

## Additional information

Tables 2,3,4,5,6 are only available in the online version of this paper.

**How to cite this article:** Zuo, X.-N. *et al.* An open science resource for establishing reliability and reproducibility in functional connectomics. *Sci. Data* 1:140049 doi: 10.1038/sdata.2014.49 (2014).

## Supplementary Material



Supplementary Information

## Figures and Tables

**Table 1:** CoRR sites and experimental design.

**Site**	**N**	**Age Range (Mean)**	**% Female**	**Retest Period**	**DOI**
*Within Session—Single Retest*					
IPCAS (Liu)—Frames of Reference [IPCAS 4]	20	21–28 (23.1)	50	44 min	http://dx.doi.org/10.15387/fcp_indi.corr.ipcas4
IPCAS (Zuo)—Intrasession [IPCAS 7]	74	6–17 (11.6)	57	8 min	http://dx.doi.org/10.15387/fcp_indi.corr.ipcas7
NYU (Castellanos) [NYU 1]	49	19.1–48 (30.3)	47	60 min	http://dx.doi.org/10.15387/fcp_indi.corr.nyu1
Southwest (Chen)—Stroop [SWU 3]	24	18–25 (20.4)	34	90 min	http://dx.doi.org/10.15387/fcp_indi.corr.swu3
Southwest (Chen)—Emotion [SWU 2]	27	18–24 (20.9)	33	32 min	http://dx.doi.org/10.15387/fcp_indi.corr.swu2

**Site**	**N**	**Age Range (Mean)**	**% Female**	**# Retests (Mean)**	**Retest Period Range (Mean Interval)**	**DOI**
*Within Session*—*Multiple Retest*						
Beijing Normal (Zang) [BNU 3]	48	18–30 (22.5)	50	2 (2)	0–8 min (4 min)	http://dx.doi.org/10.15387/fcp_indi.corr.bnu3
Berlin (Margulies) [BMB 1]	50	19.9–59.7 (30.8)	52	1 or 3 (1.4)	10–25 min (8.3 min)	http://dx.doi.org/10.15387/fcp_indi.corr.bmb1
IPCAS (Wei) [IPCAS 5]	22	18–19 (18.3)	0	1 or 2 (1.5)	10–40 min (30 min)	http://dx.doi.org/10.15387/fcp_indi.corr.ipcas5

**Site**	**N**	**Age Range (Mean)**	**% Female**	**Retest Period Range (Mean)**	**DOI**
*Between Sessions*—*Single Retest*					
IACAS (Jiang) [IACAS 1]	28	19–43 (26.4)	55	20–343 Days (75.2 Days)	http://dx.doi.org/10.15387/fcp_indi.corr.iacas1
Munich (Blautzik)—Yearly [LMU 3]	25	59–88 (69.8)	36	315–463 Days (399.6 Days)	http://dx.doi.org/10.15387/fcp_indi.corr.lmu3
Beijing Normal (He) [BNU 1]	57	19–30 (23)	47	33–55 Days (40.9 Days)	http://dx.doi.org/10.15387/fcp_indi.corr.bnu1
Beijing Normal (Liu) [BNU 2]	61	19.3–23.3 (21.3)	46	103–189 Days (160.5 Days)	http://dx.doi.org/10.15387/fcp_indi.corr.bnu2
IPCAS (Zuo)—Tai Chi [IPCAS 8]	13	50–62 (57.6)	46	367–810 Days (516 Days)	http://dx.doi.org/10.15387/fcp_indi.corr.ipcas8
Nanjing (Lu) [JHNU 1]	30	20–40 (23.3)	30	0–900 Days (202.6 Days)	http://dx.doi.org/10.15387/fcp_indi.corr.jhnu1
Southwest (Qiu) [SWU 4]	235	17–27 (20)	49	121–653 Days (302.1 Days)	http://dx.doi.org/10.15387/fcp_indi.corr.swu4
NKI (Milham) [NKI 1]	24	19–60 (34.4)	75	14 Days (14 Days)	http://dx.doi.org/10.15387/fcp_indi.corr.nki1
IPCAS (Jiang) [IPCAS 2]	35	11–15 (13.3)	65	7–59 Days (33.6 Days)	http://dx.doi.org/10.15387/fcp_indi.corr.ipcas2
MRN (Mayer, Calhoun) [MRN 1]	54	10–53 (24.9)	50	7–158 Days (109 Days)	http://dx.doi.org/10.15387/fcp_indi.corr.mrn1

**Site**	**N**	**Age Range (Mean)**	**% Female**	**# Retests (Mean)**	**Retest Period Range (Mean Interval)**	**DOI**
*Between Sessions*—*Multiple Retest*						
Hangzhou (Weng) [HNU 1]	30	20–30 (24.4)	50	9 (9)	3–40 Days (3.65 Days)	http://dx.doi.org/10.15387/fcp_indi.corr.hnu1
Pittsburgh (Luna) [UPSM 1]	100	10.1–19.7 (15.1)	48	1 or 2 (1.23)	473–1,404 Days (521 Days)	http://dx.doi.org/10.15387/fcp_indi.corr.upsm1
Munich (Blautzik)—Young Adult [LMU 1]	27	20–29 (24.3)	48	4 or 5 (4.7)	120–600 min (120 min)	http://dx.doi.org/10.15387/fcp_indi.corr.lmu1
Munich (Blautzik)—Aging [LMU 2]	40	20–79 (50.8)	45	3 (3)	150–450 min (150 min)	http://dx.doi.org/10.15387/fcp_indi.corr.lmu2
Xuanwu (Li, Lu) [XHCUMS 1]	25	36–62 (52.05)	36	4 (4)	12–197 Days (77.6 Days)	http://dx.doi.org/10.15387/fcp_indi.corr.xhcums1

**Within + Between Sessions**					**Within**	**Between**	
**Site**	**N**	**Age Range (Mean)**	**% Female**	**# Retests (Mean)**	**Retest Period Range (Mean Interval)**	**Retest Period Range (Mean Interval)**	**DOI**
IPCAS (Zuo)—3 Day [IPCAS 6]	2	21 & 25	50	44 (44)	10–22 min (11.3 min)	83–3,298 min (210 min)	http://dx.doi.org/10.15387/fcp_indi.corr.ipcas6
IBATRT (La Conte) [IBA TRT 1]	36	19–48 (26.8)	51	1 or 3 (1.4)	10 min (10 min)	51–183 Days (115.4 Days)	http://dx.doi.org/10.15387/fcp_indi.corr.ibatrt1
IPCAS (Fu) [IPCAS 1]	30	18–24 (20.9)	30	3 (3)	29 min (29 min)	5–24 Days (13.9 Days)	http://dx.doi.org/10.15387/fcp_indi.corr.ipcas1
IPCAS (Liu)—Conflict Adaptation [IPCAS 3]	36	17–25 (21)	34	1 or 3 (1.3)	40 min (40 min)	1-2 Days (1.4 Days)	http://dx.doi.org/10.15387/fcp_indi.corr.ipcas3
NYU (Di Martino) [NYU 2]	187	6.47–55.03 (20.2)	38	1, 2, 3, or 5 (1.6)	9–132 min (25.5 min)	1–203 Days (85.9 Days)	http://dx.doi.org/10.15387/fcp_indi.corr.nyu2
Utah (Anderson)—Longitudinal [Utah 1]	26	8–39 (20.2)	0	2 (2)	0 min (0 min)	733–1,187 Days (928.4 Days)	http://dx.doi.org/10.15387/fcp_indi.corr.utah1
Southwest (Chen)—Attentional Blink [SWU 1]	20	19–24 (21.5)	30	5 (5)	20 min (20 min)	20–2,900 min (1,460 min)	http://dx.doi.org/10.15387/fcp_indi.corr.swu1
Montreal (Bellec) [UM 1]	80	55–84 (65.4)	27	3 (3)	1 min (1 min)	74–194 Days (111.4 Days)	http://dx.doi.org/10.15387/fcp_indi.corr.um1
Utah Single [Utah 2]	1	39	0	100 (100)	1 min (1 min)	0–4 Days (1.75 Days)	http://dx.doi.org/10.15387/fcp_indi.corr.utah2
Wisconsin (Birn) [UWM 1]	25	21–32 (24.9)	44	2 (2)	30 min (30 min)	56–314 Days (110.4 Days)	http://dx.doi.org/10.15387/fcp_indi.corr.uwm1

**Table 2:** Imaging parameters for sMRI scans in CoRR

**Site**	**Manufacturer**	**Model**	**Headcoil**	**Field Strength**	**Sequence**	**Flip Angle [Deg]**	**Inversion Time (TI) [ms]**	**Echo Time (TE) [ms]**	**Repetition Time (TR) [ms]**	**Bandwidth per Voxel (Readout) [Hz]**	**Parallel Acquisition**	**Number of Slices**	**Orientation**	**Slice Phase Encoding Direction**	**Slice Acquisition Order**	**Slice Thickness [mm]**	**Slice Gap [mm]**	**Field of View [mm]**	**Acquisition Matrix**	**Slice In-Place Resolution [mm2]**	**Acquisition Time [min:sec]**	**Fat Suppression**	**Phase Partial Fourier**	**Notes**
Beijing Normal University 3 (BNU 3)	Siemens	TrioTim	12 Chan	3T	3D MPRAGE	7	1,100	3.39	2,530	190	Off	128	s	A-P	int+	1.33	0.6515	256	256×192	1.3×1.0	8:07	None	Off	
Berlin Mind and Brain 1 (BMB 1)	Siemens	TrioTim	12 Chan	3T	3D MPRAGE	9	900	2.98	2,300	240	Off	176	s	A-P	int+	1	0.5	256	256×256	1.0×1.0	9:50	None	Off	
Hangzhou Normal University 1 (HNU 1)	GE	Discovery MR750	8 Chan	3T	3D SPGR	8	450	Min Full	8.06	125	A2	180	s	A-P	int+	1	0	250	250×250	1.0×1.0	5:01	None	Off	
Dartmouth College (DC 1)	Philips	N/A	32 Chan	3T	3D T1-TFE	8	900	3.7	2,375	191.4	S2.5	220	a	R-L	N/A	1	N/A	240	240×187	1.0×1.0	3:06	None	N/A	Reconstructed voxels at .94×.94
Institute of Automation, Chinese Academy of Sciences 1 (IACAS 1)	GE	Signa HDx	8 Chan	3T	3D BRAVO	7	1,100	2.984	7.788	122	A2	192	s	R-L	seq+	1	0	256	256×256	1.0×1.0	5:02	None	Off	
Intrinsic Brain Activity, Test-Retest Dataset (IBATRT)	Siemens	TrioTim	12 Chan	3T	3D MPRAGE	8	900	3.02	2,600	130	G2	176	s	A-P	seq+	1	0.5	256	256×256	1.0×1.0	4:38	None	6/8	
Institute of Psychology, Chinese Academy of Sciences 1 (IPCAS 1)	Siemens	TrioTim	8 Chan	3T	MPRAGE	7	1,100	2.51	2,530	170	G2	128	s	A-P	seq+	1.3	0.65	256	256×256	1.0×1.0	5:53	None	Off	
Institute of Psychology, Chinese Academy of Sciences 2 (IPCAS 2)	Siemens	TrioTim	32 Chan	3T	MPRAGE	9	900	2.95	2,300	130	Off	160	s	A-P	seq+	1.2	0.6	240	240×226	0.9×0.9	9:14	None	Off	
Institute of Psychology, Chinese Academy of Sciences 3 (IPCAS 3)	Siemens	TrioTim	8 Chan	3T	3D MPRAGE	7	1,100	2.51	2,530	170	Off	128	s	A-P	int+	1.33		256	256×256	1.0×1.0	5:24	None	Off	
Institute of Psychology, Chinese Academy of Sciences 4 (IPCAS 4)	GE	Discovery	8 Chan	3T	3D SPGR	8	450	3.136	8,068	31.25	A2	250	s	A-P	int+	1	0	250	250×250	1.0×1.0	5:01	None	Off	
Institute of Psychology, Chinese Academy of Sciences 5 (IPCAS 5)	Siemens	TrioTim	12 Chan	3T	3D MPRAGE	7	1,100	3.5	2,530	190	G2	176	s	A-P	int+	1	0.5	256	256×256	1.0×1.0	6:03	None	Off	
Institute of Psychology, Chinese Academy of Sciences 7 (IPCAS 7)	Siemens	TrioTim	8 Chan	3T	3D MPRAGE	8	900	3.02	2,600	180	Off	176		A-P	seq+	1	0.5	256	256×256	1.0×1.0	8:19	None	6/8	
Institute of Psychology, Chinese Academy of Sciences 8 (IPCAS 8)	Siemens	TrioTim	12 Chan	3T	3D MPRAGE	7	1,100	3.39	2,530	190	Off	128	s	A-P	int+	1.3	0.65	256	256×192	1.3×1.0	8:07	None	Off	
Institute of Psychology, Chinese Academy of Sciences 6 (IPCAS 6)	Siemens	TrioTim	8 Chan	3T	3D MPRAGE	9	900	2.52	1,900	170	Off	176	s	A-P	seq+	1	0.5	250	256×246	1.0×1.0	4:17	None	Off	
University of Montreal 1 (UM 1)	Siemens	TrioTim	12 Chan	3T	3D MPRAGE	9	900	2.98	2,300	240	G2	176	s	A-P	int+	1	0.5	256	256×256	1.0×1.0	5:12	None	Off	
Mind Research Network (MRN 1)	Siemens	TrioTim	12 Chan	3T	3D MEMPR	7	1,200	1.64/3.5/5.36/7.22/9.08	2,530	651	G2	192	s oblique	A-P	int+	1	0.5	256	256×256	1.0×1.0	6:03	None	Off	
Ludwig-Maximilians-University 2 (LMU 2)	Siemens	Verio	12 Chan	3T	3D MPRAGE	9	900	3.06	2,400	230	G2	160	s	A-P	int+	1	0.5	256	256×246	1.0×1.0	4:45	None	7/8	
Ludwig-Maximilians-University 1 (LMU 1)	Philips	Achieva	32 Chan	3T	3D T1-TFE	8	900	N/A	2,375	191.5	S2/2.5	220	a	R-L	seq+	1	0	240	240×187	1.0×1.0	3:06	None	None	Reconstructed voxels at .94×.94
Ludwig-Maximilians-University 3 (LMU 3)	Siemens	TrioTim	12 Chan	3T	3D MPRAGE	9	900	3.06	2,400	230	G2	256	s	A-P	int+	1	0.5	256	256×246	1.0×1.0	4:45	None	7/8	
Jinling Hospital, Nanjing University 1 (JHNU 1)	Siemens	TrioTim	8 Chan	3T	3D MPRAGE	9	900	2.98	2,300	240	Off	176	s	A-P	seq+	1	0	256	256×256	1.0×1.0	9:50	None	Off	
Nathan Kline Institute 1 (NKI 1)	Siemens	TrioTim	32 Chan	3T	3D MPRAGE	9	900	2.52	1,900	170	G2	176	s	A-P	seq+	1	0.5	250	256×246	1.0×1.0	4:18	None	Off	
New York University 2 (NYU 2)	Siemens	Allegra	1 Chan	3T	3D MPRAGE	7	1,100	3.25	2,530	200	Off	128	s	A-P	seq+	1.3	0.65	256	256×192	1.3×1.0	8:07	None	Off	
New York University 1 (NYU 1)	Siemens	Allegra	1 Chan	3T	MPRAGE	8	900	3.93	2,500	N/A	N/A	176	N/A	N/A	N/A	1	N/A	256	256×256	1.0×1.0	N/A	N/A	N/A	
University of Pittsburgh School of Medicine (UPSM)	Siemens	TrioTim	12 Chan	3T	3D MPRAGE	8	1,050	3.43	2,100	240	G2	192	a oblique	R-L	int+	1	0.5	256	256×256	1.0×1.0	3:59	None	Off	
Southwest University 1 (SWU 1)	Siemens	TrioTim	8 Chan	3T	3D MPRAGE	9	900	2.52	1,900	170	G2	176	s	A-P	seq+	1	0.5	250	256×246	1.0×1.0	4:18	None	Off	
Southwest University 3 (SWU 3)	Siemens	TrioTim	8 Chan	3T	3D MPRAGE	9	900	2.52	1,900	170	G2	176	s	A-P	seq+	1	0.5	250	256×246	1.0×1.0	4:18	None	Off	
Southwest University 2 (SWU 2)	Siemens	TrioTim	8 Chan	3T	3D MPRAGE	9	900	2.52	1,900	170	G2	176	s	A-P	seq+	1	0.5	250	256×246	1.0×1.0	4:18	None	Off	
Southwest University 4 (SWU 4)	Siemens	TrioTim	8 Chan	3T	3D MPRAGE	9	900	2.52	1,900	170	G2	176	s	A-P	seq+	1	0.5	256	256×256	1.0×1.0	4:26	None	Off	
Beijing Normal University 1 (BNU 1)	Siemens	TrioTim	12 Chan	3T	3D MPRAGE	7	1,100	3.39	2,530	256	Off	144	s	A-P	int+	1.3	0.65	256	256×192	1.3×1.0	8:07	None	Off	
Beijing Normal University 2 (BNU 2) (Test)	Siemens	TrioTim	12 Chan	3T	3D MPRAGE	7	1,100	3.39	2,530	256	Off	128	s	A-P	int+	1.3	0.65	256	256×192	1.3×1.0	8:07	None	Off	
Beijing Normal University 2 (BNU 2) (Retest)	Siemens	TrioTim	12 Chan	3T	3D MPRAGE	7	1,100	3.45	2,530	256	Off	176	s	A-P	int+	1	0.5	256	256×256	1.0×1.0	10:49	None	Off	
University of Utah 1 (Utah 1)	Siemens	TrioTim	12 Chan	3T	3D MPRAGE	9	900	2.91	2,300	240	Off	160	s	A-P	int+	1.2	0.6	256	256×256	1.0×1.0	9:14	None	Off	
University of Utah 2 (Utah 2)	Siemens	TrioTim	12 Chan	3T	3D MPRAGE	9	900	2.91	2,300	240	Off	160	s	A-P	int+	1.2	0.6	256	256×256	1.0×1.0	9:14	None	Off	
University of Washington—Madison 1 (UWM 1)	GE	Discovery	8 Chan	3T	3D MPRAGE	12	450	3.18	8.13	244	Off	160	a	R-L	Simultaneous (3D)	1	0	256	256×256	1.0×1.0	7:30	None	Off	
Xuanwu Hospital, Capital University of Medical Sciences 1 (XHCUMS 1)	Siemens	TrioTim	12 Chan	3T	3D MPRAGE	9	800	2.15	1,600	200	Off	176	s oblique	A-P	seq+	1	0.5	256	256×256	1.0×1.0	5:09	None	6/8	

**Table 3:** Imaging parameters for rfMRI scans in CoRR

**Site**	**Manufacturer**	**Model**	**Headcoil**	**Field Strength**	**Sequence**	**Flip Angle [Deg]**	**Echo Time (TE) [ms]**	**Repetition Time (TR) [ms]**	**Bandwidth per Voxel (Readout) [Hz]**	**Parallel Acquisition**	**Number of Slices**	**Orientation**	**Slice Phase Encoding Direction**	**Slice Acquisition Order**	**Slice Thickness [mm]**	**Slice Gap [mm]**	**Field of View [mm]**	**Acquisition Matrix**	**Slice In-Place Resolution [mm2]**	**Number of Measurements**	**Acquisition Time [min:sec]**	**Fat Suppression**	**Prospective Motion Correction**	**Retrospective Motion Correction**	**Notes**
Beijing Normal University 3 (BNU 3)	Siemens	TrioTim	12 Chan	3T	EPI	90	30	2,000	2,520	Off	34	a	A-P	int+	3.5	0.7	200	64×64	3.5×3.5	150	8:06	Yes	No	No	
Berlin Mind and Brain 1 (BMB 1)	Siemens	TrioTim	12 Chan	3T	EPI	90	30	2,300	2,232	Off	34	a	A-P	int+	4	0	192	64×64	3.0×3.0	200	7:45	Yes	No	No	
Hangzhou Normal University 1 (HNU 1)	GE	Discovery MR750	8 Chan	3T	EPI	90	30	2,000	3437.5	On	43	a	A-P	int+	3.4	0	220	64×64	3.4×3.4	300	10:00	Yes	No	No	
Dartmouth College (DC 1)	Philips	N/A	32 Chan	3T	EPI	90	35	2,500	3,625	S2	36	a	A-P	N/A	3.5	0.5	240	80×80	3.0×3.0	120	5:10	Yes	No	N/A	
Institute of Automation, Chinese Academy of Sciences 1 (IACAS 1)	GE	Signa HDx	8 Chan	3T	EPI	90	30	2,000	7812.5	Off	32	N/A	R-L	int+	4	0.6	220	64×64	3.4×3.4	240	8:00	No	N/A	N/A	
Intrinsic Brain Activity, Test-Retest Dataset (IBATRT)	Siemens	TrioTim	12 Chan	3T	EPI	90	30	1,750	2,442	Off	29	a	A-P	seq+	3.6	0.36	220	64×64	3.4×3.4	343	10:04	Yes	No	No	
Institute of Psychology, Chinese Academy of Sciences 1 (IPCAS 1)	Siemens	TrioTim	8 Chan	3T	EPI	90	30	2,000	2,232	Off	32	a	A-P	int+	4	0.8	256	64×64	4.0×4.0	205	6:54	Yes	No	N/A	
Institute of Psychology, Chinese Academy of Sciences 2 (IPCAS 2)	Siemens	TrioTim	32 Chan	3T	EPI	90	30	2,500	2,232	Off	32	a	A-P	int+	3	0.99	240	64×64	3.8×3.8	212	8:57	Yes	Yes	No	
Institute of Psychology, Chinese Academy of Sciences 3 (IPCAS 3)	Siemens	TrioTim	8 Chan	3T	EPI	90	30	2,000	2,232	Off	64	a	A-P	int+	3	0.99	220	64×64	3.4×3.4	180	6:00	Yes	No	No	
Institute of Psychology, Chinese Academy of Sciences 4 (IPCAS 4)	GE	Discovery MR750	8 Chan	3T	EPI	90	30	2,000	250	Off	37	a	A-P	int+	3.5	0	224	64×64	3.5×3.5	180	6:04	Yes	No	No	
Institute of Psychology, Chinese Academy of Sciences 5 (IPCAS 5)	Siemens	TrioTim	12 Chan	3T	EPI	90	30	2,000	2,298	Off	33	c	F-H	int+	5	0	200	64×64	3.1×3.1	170	5:44	Yes	No	No	
Institute of Psychology, Chinese Academy of Sciences 7 (IPCAS 7)	Siemens	TrioTim	8 Chan	3T	EPI	80	30	2,500	2,240	Off	38	a	A-P	int+	3	0.33	216	72×72	3.0×3.0	184	7:45	Yes	No	No	
Institute of Psychology, Chinese Academy of Sciences 8 (IPCAS 8)	Siemens	TrioTim	12 Chan	3T	EPI	90	30	2,000	2,520	Off	33	a	A-P	int+	3	0.9	220	64×64	3.4×3.4	240	8:06	Yes	Yes	No	
Institute of Psychology, Chinese Academy of Sciences 6 (IPCAS 6)	Siemens	TrioTim	8 Chan	3T	EPI	90	30	2,500	2,298	Off	25	a	A-P	int+	3.5	3.5	224	64×64	3.5×3.5	242	10:05	Yes	No	No	
University of Montreal 1 (UM 1)	Siemens	TrioTim	12 Chan	3T	EPI	90	30	2,000	2,442	Off	32	a	A-P	seq-	4	0	256	64×64	4.0×4.0	150	5:04	Yes	No	No	
Mind Research Network (MRN 1)	Siemens	TrioTim	12 Chan	3T	EPI	75	29	2,000	2,170	Off	33	a oblique	A-P	int+	3.5	1.05	240	64×64	3.8×3.8	150	5:04	Yes	No	No	
Ludwig-Maximilians-University 2 (LMU 2)	Siemens	Verio	12 Chan	3T	EPI	80	30	3,000	2,232	Off	28	a	A-P	int+	4	0.4	192	64×64	3.0×3.0	120	6:06	Yes	No	Yes	
Ludwig-Maximilians-University 1 (LMU 1)	Philips	Achieva	32 Chan	3T	EPI	90	30	2,500	2,032	S3	52	a	A-P	seq+	3	0	224×233	76×79	2.95×2.95	180	7:35	Yes	Yes	N/A	Data Reconstructed at 1.65×1.65 in plane resolution
Ludwig-Maximilians-University 3 (LMU 3)	Siemens	TrioTim	12 Chan	3T	EPI	80	30	3,000	2,232	Off	36	a	A-P	int+	4	0.4	192	64×64	3.0×3.0	120	6:06	Yes	No	No	
Jinling Hospital, Nanjing University 1 (JHNU 1)	Siemens	TrioTim	8 Chan	3T	EPI	90	30	2,000	2,230	2	30	a	A-P	int+	4	0.4	240	64×64	3.75×3.75	250	8:20	Yes	No	No	
Nathan Kline Institute 1 (NKI 1) (2500)	Siemens	TrioTim	32 Chan	3T	EPI	80	30	2,500	2,240	Off	38	a	A-P	int+	3	0.33	216	72×72	3.0×3.0	120	5:05	Yes	No	No	
Nathan Kline Institute 1 (NKI 1) (1400)	Siemens	TrioTim	32 Chan	3T	EPI	65	30	1,400	1,786	Off	64	a	A-P	int+	2	0	224	112×112	2.0×2.0	404	9:35	Yes	No	No	
Nathan Kline Institute 1 (NKI 1) (645)	Siemens	TrioTim	32 Chan	3T	EPI	60	30	645	2,598	Off	40	a	A-P	int+	3	0	222	74×74	3.0×3.0	900	9:46	Yes	No	No	
New York University 2 (NYU 2)	Siemens	Allegra	1 Chan	3T	EPI	90	15	2,000	3,906	Off	33	a oblique	R-L	int+	4	0	240	80×80	3.0×3.0	180	6:00	Yes	No	N/A	
New York University 1 (NYU 1)	Siemens	Allegra	1 Chan	3T	EPI	90	25	2,000	N/A	N/A	39	N/A	N/A	N/A	3	N/A	192	64×64	3.0×3.0	197	6:34	N/A	N/A	N/A	
University of Pittsburgh School of Medicine (UPSM)	Siemens	TrioTim	12 Chan	3T	EPI	70	29	1,500	2,694	G2	29	a oblique	P-A	seq+	4	0	200	64×64	3.1×3.1	200	5:06	Yes	No	Yes	
Southwest University 1 (SWU 1)	Siemens	TrioTim	8 Chan	3T	EPI	90	30	2,000	2,232	Off	33	a	A-P	int+	3	0.6	200	64×64	3.1×3.1	240	8:06	Yes	Yes	No	
Southwest University 3 (SWU 3)	Siemens	TrioTim	8 Chan	3T	EPI	90	30	2,000	2,232	Off	32	a oblique	A-P	int+	3	0.99	220	64×64	3.4×3.4	242	8:08	Yes	Yes	No	
Southwest University 2 (SWU 2)	Siemens	TrioTim	8 Chan	3T	EPI	90	30	2,000	2,232	Off	32	a oblique	A-P	int+	3	0.99	220	64×64	3.4×3.4	300	10:04	Yes	Yes	No	
Southwest University 4 (SWU 4)	Siemens	TrioTim	8 Chan	3T	EPI	90	30	2,000	2,232	Off	32	a	A-P	int+	3	1	220	64×64	3.4×3.4	242	8:06	Yes	Yes	No	
Beijing Normal University 1 (BNU 1)	Siemens	TrioTim	12 Chan	3T	EPI	90	30	2,000	2,520	Off	33	a	A-P	int+	3.5	0.7	200	64×64	3.1×3.1	200	6:46	Yes	No	No	
Beijing Normal University 2 (BNU 2) (Test)	Siemens	TrioTim	12 Chan	3T	EPI	90	30	2,000	2,520	Off	33	a	A-P	int+	3	0.6	200	64×64	3.1×3.1	240	8:06	Yes	No	No	
Beijing Normal University 2 (BNU 2) (Retest)	Siemens	TrioTim	12 Chan	3T	EPI	90	30	1,500	2,520	Off	25	a	A-P	int+	4	0.8	200	64×64	3.1×3.1	420	10:36	Yes	No	Yes	
University of Utah 1 (Utah 1)	Siemens	TrioTim	12 Chan	3T	EPI	90	28	2,000	2,894	Off	40	a	A-P	int+	3	0.3	220	64×64	3.4×3.4	240	8:06	Yes	No	Yes	
University of Utah 2 (Utah 2)	Siemens	TrioTim	12 Chan	3T	EPI	90	28	2,000	2,894	Off	40	a	A-P	int+	3	0.3	220	64×64	3.4×3.4	240	8:06	Yes	No	Yes	
University of Washington—Madison 1 (UWM 1)	GE	Discovery MR750	8 chan	3T	EPI	60	25	2,600	N/A	Off	40	N/A	A-P	int+	3.5	0	224	64×64	3.5×3.5	231	10:01	No (Spectral Spatial RF pulse)	N/A	N/A	
Xuanwu Hospital, Capital University of Medical Sciences 1 (XHCUMS 1)	Siemens	TrioTim	12 Chan	3T	EPI	90	30	3,000	2,232	Off	43	a oblique	A-P	int+	3	0.48	192	64×64	3.0×3.0	124	6:20	Yes	No	N/A	

**Table 4:** Imaging parameters for dMRI scans in CoRR

**Site**	**Manufacturer**	**Model**	**Sequence**	**Headcoil**	**Field Strength**	**Flip Angle [Deg]**	**Echo Time (TE) [ms]**	**Repetition Time (TR) [ms]**	**Bandwidth per Voxel (Readout) [Hz]**	**Parallel Acquisition**	**Number of Slices**	**Orientation**	**Slice Phase Encoding Direction**	**Slice Acquisition Order**	**Slice Thickness [mm]**	**Slice Gap [mm]**	**Field of View [mm]**	**Acquisition Matrix**	**Slice In-Place Resolution [mm** ^**2**^]	**Number of Measurements**	**Acquisition Time [min:sec]**	**Fat Suppression**	**Phase Partial Fourier**	**Number of Directions**	**Number of B Zeros**	**B Value(s) [s/mm** ^**2**^]	**Averages**	**Notes**
Beijing Normal University 3 (BNU 3)	Siemens	TrioTim	EPI		3T	N/A	104	7,200	1,396	G2	49	a	A-P	int+	2.5	0	230	128×128	1.8×1.8	65	8:11	Yes	None	64	1	1,000	1	
Hangzhou Normal University 1 (HNU 1)	GE						Min	8,600			68		R-L	int+	1.5	0	192	128×128	1.5×1.5	33		Yes		30		1,000		
Institute of Psychology, Chinese Academy of Sciences (IPCAS 1)	Siemens	TrioTim																		62				62				
Institute of Psychology, Chinese Academy of Sciences (IPCAS 2)																				39								
Institute of Psychology, Chinese Academy of Sciences (IPCAS 8)	Siemens	TrioTim	EPI		3T		104	6,600	1,396	G2	45	a	A-P	int+	3	0	230	128×128	1.8×1.8	65	7:30	Yes	None	64	1	1,000	1	
Mind Research Network 1 (MRN 1)	Siemens	TrioTim	EPI		3T	N/A	84	9,000	1,562	G2	72	a	A-P	int+	2	0	256	128×128	2.0×2.0	35	5:42	Yes	6/8	35	0	800	1	
Nathan Kline Institute 1 (NKI 1)	Siemens	TrioTim	EPI		3T	90	85	2,400	1,814	Off	64	a	A-P	int+	2	0	212	106×106	2.0×2.0	137	5:58	Yes	6/8	137	0	1,500	1	
Southwest University 4 (SWU 4)	Siemens	TrioTim																		93								
Beijing Normal University 1 (BNU 1)	Siemens	TrioTim	EPI		3T		89	8,000	1,562	G2	62	a	A-P	int+	2.2	0	282	128×128	2.2×2.2	31	4:34	Yes	6/8	30	1	1,000	1	
Xuanwu Hospital, Capital University of Medical Sciences (XHCUMS 1)	Siemens	TrioTim	EPI		3T		83	8,000	1,396	G2	64	a	A-P	int+	2	0	256	128×128	2.0×2.0	65	9:06	Yes	6/8	64	1	700	1	

**Table 5:** Phenotypic protocols in CoRR

**CoRR Data Legend**	**COLUMN LABEL**	**DESCRIPTION**	**DATA TYPE**	**REQUIREMENT LEVEL**
	SUBID	INDI Subject ID	integer	core
	AGE_AT_SCAN_1	Age at scan session 1 in years (1 decimal place)	float	core
	SEX	sex (1: female, 2: male)	integer	core
	DSM_IV_TR	DSM-based Psychiatric Diagnosis (CPT Code)	integer	optional
	FIQ	Full-scale IQ	integer	optional
	VIQ	Verbal IQ	integer	optional
	PIQ	Peformance IQ	integer	optional
	BMI	Body Mass Index	float	optional
	RESTING STATE_INSTRUCTION	Instruction	string	core
	VISUAL_STIMULATION_CONDITION	Visual stimulation for rest (1: fixation, 2: blank screen, 3: word, 4: eyes closed, 5: other)	integer	core
	RETEST DESIGN	1: Within Session, 2: Between Session, 3: Within + Between	integer	core
baseline	PRECEDING_CONDITION	0: No active task, 1: active task, 2: music listening, 3: video watching, 4: unknown	integer	core
	TIME_OF_DAY	0[0-5:59], 1[6:00-11:59], 2[12:00-17:59], 3[18:00-23:59]	integer	preferred
	SATIETY	0: unknown, 1: post-prandial, 2: fasting	integer	preferred
	LMP	Number of days since start of last menstrual period (−1: male, 0: unknown)	integer	preferred
retest 1	RETEST DURATION	Time since baseline	real	core
	RETEST_UNITS	m: min, d: days	string	core
	PRECEDING_CONDITION	0: No active task, 1: active task, 2: music listening, 3: video watching, 4: unknown	integer	core
	TIME_OF_DAY	0[0-5:59], 1[6:00-11:59], 2[12:00-17:59], 3[18:00-23:59]	integer	preferred
	SATIETY	0: unknown, 1: post-prandial, 2: fasting	integer	preferred
	LMP	Number of days since start of last menstrual period (−1: male, 0: unknown)	integer	preferred
retest 2	RETEST DURATION	Time since baseline	real	core
	RETEST_UNITS	m: min, d: days	string	core
	PRECEDING_CONDITION	0: No active task, 1: active task, 2: music listening, 3: video watching, 4: unknown	integer	core
	TIME_OF_DAY	0[0-5:59], 1[6:00-11:59], 2[12:00-17:59], 3[18:00-23:59]	integer	preferred
	SATIETY	0: unknown, 1: post-prandial, 2: fasting	integer	preferred
	LMP	Number of days since start of last menstrual period (−1: male, 0: unknown)	integer	preferred
retest 3	RETEST DURATION	Time since baseline	real	core
	RETEST_UNITS	m: min, d: days	string	core
	PRECEDING_CONDITION	0: No active task, 1: active task, 2: music listening, 3: video watching, 4: unknown	integer	core
	TIME_OF_DAY	0[0-5:59], 1[6:00-11:59], 2[12:00-17:59], 3[18:00-23:59]	integer	preferred
	SATIETY	0: unknown, 1: post-prandial, 2: fasting	integer	preferred
	LMP	Number of days since start of last menstrual period (−1: male, 0: unknown)	integer	preferred
retest 4	RETEST DURATION	Time since baseline	real	core
	RETEST_UNITS	m: min, d: days	string	core
	PRECEDING_CONDITION	0: No active task, 1: active task, 2: music listening, 3: video watching, 4: unknown	integer	core
	TIME_OF_DAY	0[0-5:59], 1[6:00-11:59], 2[12:00-17:59], 3[18:00-23:59]	integer	preferred
	SATIETY	0: unknown, 1: post-prandial, 2: fasting	integer	preferred
	LMP	Number of days since start of last menstrual period (-1: male, 0: unknown)	integer	preferred
retest 5	RETEST DURATION	Time since baseline	real	core
	RETEST_UNITS	m: min, d: days	string	core
	PRECEDING_CONDITION	0: No active task, 1: active task, 2: music listening, 3: video watching, 4: unknown	integer	core
	TIME_OF_DAY	0[0-5:59], 1[6:00-11:59], 2[12:00-17:59], 3[18:00-23:59]	integer	preferred
	SATIETY	0: unknown, 1: post-prandial, 2: fasting	integer	preferred
	LMP	Number of days since start of last menstrual period (−1: male, 0: unknown)	integer	preferred
retest 6	RETEST DURATION	Time since baseline	real	core
	RETEST_UNITS	m: min, d: days	string	core
	PRECEDING_CONDITION	0: No active task, 1: active task, 2: music listening, 3: video watching, 4: unknown	integer	core
	TIME_OF_DAY	0[0-5:59], 1[6:00-11:59], 2[12:00-17:59], 3[18:00-23:59]	integer	preferred
	SATIETY	0: unknown, 1: post-prandial, 2: fasting	integer	preferred
	LMP	Number of days since start of last menstrual period (−1: male, 0: unknown)	integer	preferred
retest 7	RETEST DURATION	Time since baseline	real	core
	RETEST_UNITS	m: min, d: days	string	core
	PRECEDING_CONDITION	0: No active task, 1: active task, 2: music listening, 3: video watching, 4: unknown	integer	core
	TIME_OF_DAY	0[0-5:59], 1[6:00-11:59], 2[12:00-17:59], 3[18:00-23:59]	integer	preferred
	SATIETY	0: unknown, 1: post-prandial, 2: fasting	integer	preferred
	LMP	Number of days since start of last menstrual period (-1: male, 0: unknown)	integer	preferred
retest 8	RETEST DURATION	Time since baseline	real	core
	RETEST_UNITS	m: min, d: days	string	core
	PRECEDING_CONDITION	0: No active task, 1: active task, 2: music listening, 3: video watching, 4: unknown	integer	core
	TIME_OF_DAY	0[0-5:59], 1[6:00-11:59], 2[12:00-17:59], 3[18:00-23:59]	integer	preferred
	SATIETY	0: unknown, 1: post-prandial, 2: fasting	integer	preferred
	LMP	Number of days since start of last menstrual period (−1: male, 0: unknown)	integer	preferred
retest 9	RETEST DURATION	Time since baseline	real	core
	RETEST_UNITS	m: min, d: days	string	core
	PRECEDING_CONDITION	0: No active task, 1: active task, 2: music listening, 3: video watching, 4: unknown	integer	core
	TIME_OF_DAY	0[0-5:59], 1[6:00-11:59], 2[12:00-17:59], 3[18:00-23:59]	integer	preferred
	SATIETY	0: unknown, 1: post-prandial, 2: fasting	integer	preferred
	LMP	Number of days since start of last menstrual period (−1: male, 0: unknown)	integer	preferred

**Table 6:** Descriptive statistics for common derivatives

**site**	**falff_50_mean**	**falff_50_std**	**falff_75_mean**	**falff_75_std**	**falff_90_mean**	**falff_90_std**	**reho_50_mean**	**reho_50_std**	**reho_75_mean**	**reho_75_std**	**reho_90_mean**	**reho_90_std**	**vmhc_50_mean**	**vmhc_50_std**	**vmhc_75_mean**	**vmhc_75_std**	**vmhc_90_mean**	**vmhc_90_std**
BMB_1	0.67187915	0.01095439	0.71275284	0.01375119	0.75674746	0.01764103	0.11483976	0.02234383	0.17367675	0.03054432	0.23555456	0.03477065	0.40005245	0.05349399	0.59060775	0.05345469	0.74120895	0.04415967
UPSM_1	0.53848629	0.01365502	0.58279827	0.01723196	0.63421968	0.02156525	0.10928463	0.01861684	0.15879749	0.02548246	0.21595553	0.03151972	0.36652558	0.08059337	0.55897114	0.07633087	0.72308106	0.05822021
LMU_1	0.68131285	0.07205763	0.71701862	0.07185605	0.75380735	0.07318461	0.19503535	0.01811093	0.26123735	0.02698287	0.34628153	0.0460762	0.36543758	0.08438397	0.57967558	0.10305263	0.75077383	0.07739737
LMU_2	0.75128582	0.01154888	0.78492683	0.01157899	0.81470764	0.01302299	0.08188113	0.07398937	0.11549643	0.07391351	0.15802806	0.07467288	0.27874734	0.09491569	0.47506016	0.09555446	0.6654087	0.07868861
LMU_3	0.75300309	0.01130565	0.78767158	0.01167477	0.81832079	0.0126824	0.08800187	0.01217619	0.12622061	0.020186	0.17269627	0.02807193	0.3391815	0.06357559	0.53838804	0.06600959	0.70707509	0.05175325
HNU_1	0.65986927	0.02010203	0.72070762	0.02451698	0.77651227	0.02363225	0.2038152	0.03514323	0.29749165	0.04497476	0.38325751	0.05052365	0.4588192	0.05497453	0.63538063	0.04934746	0.77230292	0.03885562
IPCAS_1	0.67044235	0.016092	0.73265578	0.02054316	0.78949274	0.02176515	0.16103934	0.01787039	0.23594238	0.02272643	0.30939742	0.0268952	0.46294367	0.04570625	0.64824828	0.03803198	0.7883282	0.0275335
IPCAS_8	0.62967971	0.01925352	0.67052505	0.02387977	0.71691484	0.02876108	0.09530096	0.01353402	0.14184759	0.02166288	0.1948324	0.02725064	0.36661959	0.07089762	0.55197346	0.07581143	0.70837986	0.06042681
IPCAS_3	0.63595724	0.01076589	0.68743886	0.01482221	0.74423368	0.01892027	0.11979874	0.01402373	0.18396178	0.01845976	0.24977681	0.02327644	0.40684854	0.06845373	0.60042805	0.06228979	0.75188672	0.04717628
BNU_2	0.60229242	0.02920188	0.65412869	0.02554877	0.71380426	0.02530338	0.11757821	0.02282137	0.17708686	0.03426596	0.23930029	0.04184171	0.39161112	0.06085424	0.57577467	0.06092372	0.72323258	0.05214133
Utah_2	0.39387042	0.00795506	0.43954022	0.01013165	0.48927946	0.01422875	0.09003811	0.0073556	0.13869799	0.01255276	0.199258	0.01605418	0.29095939	0.03879641	0.51411575	0.04272958	0.696097	0.03564367
IPCAS_2	0.72243191	0.0181438	0.7615492	0.02058029	0.799527	0.02245114	0.11424405	0.01332137	0.17020604	0.01854308	0.22841676	0.02230091	0.38997572	0.0479311	0.57226243	0.04429783	0.72028097	0.03543431
IPCAS_7	0.70453551	0.01044921	0.74220274	0.01239562	0.78069057	0.01499633	0.11302486	0.01391228	0.16486068	0.01887637	0.22047912	0.02306602	0.44019481	0.05222028	0.61713186	0.04193198	0.75632372	0.0310469
IPCAS_4	0.61859584	0.00500133	0.67050929	0.00733894	0.73398443	0.00864194	0.15711392	0.01341702	0.24042446	0.01581406	0.32192849	0.01555375	0.33438623	0.04830237	0.53106513	0.03968627	0.70159497	0.02432706
IBA_TRT	0.61697087	0.01698959	0.67181163	0.02379427	0.73267667	0.02564145	0.15428888	0.01943454	0.22296525	0.02621619	0.28959599	0.03162183	0.49319222	0.06024092	0.66792044	0.05406984	0.79630123	0.04112627
NYU_1	0.60403584	0.00560845	0.63578872	0.00704961	0.66602103	0.01062687	0.06655346	0.00876404	0.08898365	0.01330434	0.11775377	0.01752209	0.24062456	0.06428724	0.4098162	0.07451068	0.58509115	0.06925713
SWU_3	0.64630782	0.01126605	0.69593592	0.0152442	0.75454278	0.01890379	0.124959	0.01111077	0.18522821	0.01390574	0.24769718	0.01560898	0.42335421	0.05311485	0.59702631	0.05107783	0.73650282	0.04191661
JHNU_1	0.65301786	0.01257395	0.70823791	0.01853576	0.7656215	0.02257707	0.14548168	0.01738676	0.21816082	0.02548086	0.29086169	0.03104211	0.43962768	0.04908573	0.62718721	0.04797892	0.76920497	0.04042416
IPCAS_6	0.70658553	0.0123221	0.74488307	0.01826713	0.78379522	0.02078967	0.10545752	0.01273462	0.15660753	0.02326668	0.21339069	0.03337394	0.34452537	0.04373743	0.53229765	0.04768446	0.69326661	0.04242879
IPCAS_5	0.64256233	0.01487854	0.69059087	0.02347256	0.74449512	0.02796	0.11943758	0.0155287	0.17868678	0.02501478	0.23846551	0.03047227	0.4077564	0.05064864	0.59247696	0.05081532	0.73817232	0.04471268
SWU_2	0.64974047	0.01289791	0.70310073	0.0188145	0.76135469	0.02277764	0.12797104	0.01927335	0.19042776	0.02500273	0.25444691	0.03008468	0.45193177	0.05525688	0.63140079	0.05077888	0.76819185	0.04285344
BNU_1	0.63211946	0.00972767	0.67600309	0.01378115	0.72653647	0.01881311	0.10428446	0.01308989	0.15421598	0.01991249	0.21087879	0.02547435	0.35300216	0.05553608	0.53670762	0.05706393	0.69495155	0.04838126
SWU_4	0.64154444	0.01429541	0.69082036	0.0203162	0.74711214	0.02426893	0.11653525	0.01383388	0.17615494	0.01984331	0.2391432	0.02410923	0.39455079	0.06615914	0.58018861	0.06404032	0.73106233	0.0485205
XHCUMS_1	0.74545799	0.0078227	0.77938982	0.0088829	0.81059326	0.0107497	0.07256239	0.01079506	0.10624958	0.01963938	0.1511244	0.02958381	0.30188529	0.06824965	0.50331368	0.08017147	0.68419305	0.07082655
IACAS_1	0.6895231	0.0300066	0.75245037	0.03322049	0.8018579	0.0332941	0.24198074	0.02482892	0.33039185	0.03241646	0.41397883	0.04006509	0.52029517	0.06943379	0.69257685	0.05949973	0.81463929	0.0401705
UWM_1	0.73885091	0.02085548	0.78182404	0.0217158	0.82110711	0.02122855	0.18033792	0.02375627	0.26637009	0.03235537	0.34847208	0.03830241	0.43066953	0.06329518	0.62068197	0.05952193	0.76718269	0.04542059
Utah_1	0.43180294	0.01134049	0.47441855	0.01467621	0.52603847	0.02023981	0.09954567	0.01276878	0.14814368	0.01833839	0.20506961	0.02249829	0.32673934	0.06492514	0.53635086	0.06411349	0.71313155	0.04848159
MRN_1	0.65478119	0.01662275	0.7120571	0.02363398	0.76604944	0.02670331	0.15111643	0.02284248	0.2216864	0.02877418	0.29443578	0.03425655	0.48563286	0.06569892	0.67372623	0.0542015	0.80905918	0.0375586
BNU_3	0.62995743	0.01655295	0.6739848	0.02082506	0.72541729	0.02575076	0.1084434	0.01530951	0.16294329	0.02273817	0.222565	0.02686687	0.36913241	0.0628315	0.5559278	0.06363683	0.71159108	0.05368372
NYU_2	0.60924566	0.00834139	0.64618221	0.01101985	0.68359507	0.01529646	0.09548649	0.01852433	0.13417889	0.02440248	0.17756895	0.02930165	0.31585856	0.07920283	0.5005881	0.07993809	0.66899026	0.06534853
UM_1	0.64465695	0.01904965	0.69641997	0.02274992	0.7489135	0.02579444	0.18495672	0.0263751	0.25570424	0.03319021	0.32440271	0.03714822	0.5210892	0.06289882	0.7000433	0.05041404	0.8275942	0.03329554
SWU_1	0.63444905	0.01143149	0.6768667	0.01638089	0.72632754	0.02163575	0.12247162	0.01482465	0.17643713	0.02030604	0.23413699	0.02418896	0.38041606	0.05010822	0.55571521	0.0510355	0.70154697	0.04606569
nki_rest_645	0.42075741	0.03601592	0.51325902	0.04760785	0.62189991	0.05329492	0.16792067	0.02582188	0.26346495	0.03860451	0.35137484	0.04565719	0.53317793	0.08586873	0.72076799	0.07001122	0.83808126	0.04968654
nki_rest_1400	0.5329489	0.0157548	0.58441685	0.02376856	0.6584884	0.03356512	0.13901774	0.01651447	0.21431023	0.03082392	0.30258362	0.04254909	0.47995716	0.08000626	0.68036714	0.06892006	0.81266311	0.05198713
nki_rest_2500	0.69885965	0.01782518	0.74602209	0.02094921	0.78988815	0.02365705	0.10835936	0.01536915	0.16690148	0.02466222	0.23075883	0.0325903	0.45334	0.06787562	0.65129144	0.05635185	0.79011133	0.03912288
