# Supplementary material for: An open science resource for establishing reliability and reproducibility in functional connectomics
Source: Sci Data. 2014 Dec 9;1:140049. doi: 10.1038/sdata.2014.49 (PMC4421932; doi:10.1038/sdata.2014.49)
Supplement: Supplementary Information [file sdata201449-s2.pdf]

An Open Science Resource for Establishing Reliability and Reproducibility in Functional Connectomics: Supplementary Information

<u>Table of Contents</u>

Supplementary Figure 1 ……………………………………….. 2
Quality assessment metrics of sMRI scans in CoRR (N = 3357)

Supplementary Figure 2 ……………………………………….. 3
Quality assessment metrics of rfMRI scans in CoRR (N = 5093)

Supplementary Figure 3 ……………………………………….. 4
Quality assessment on head motion of rfMRI scans in CoRR (N = 3357)

Supplementary Figure 4 ……………………………………….. 5
Test-retest plots of site-related mean and coefficient of variation across common derivatives (N = 1019) for the 75th percentile (mean, 1st column; coefficient of variation, 2nd column) and the 90th percentile (mean, 3rd column; coefficient of variation, 4th column)

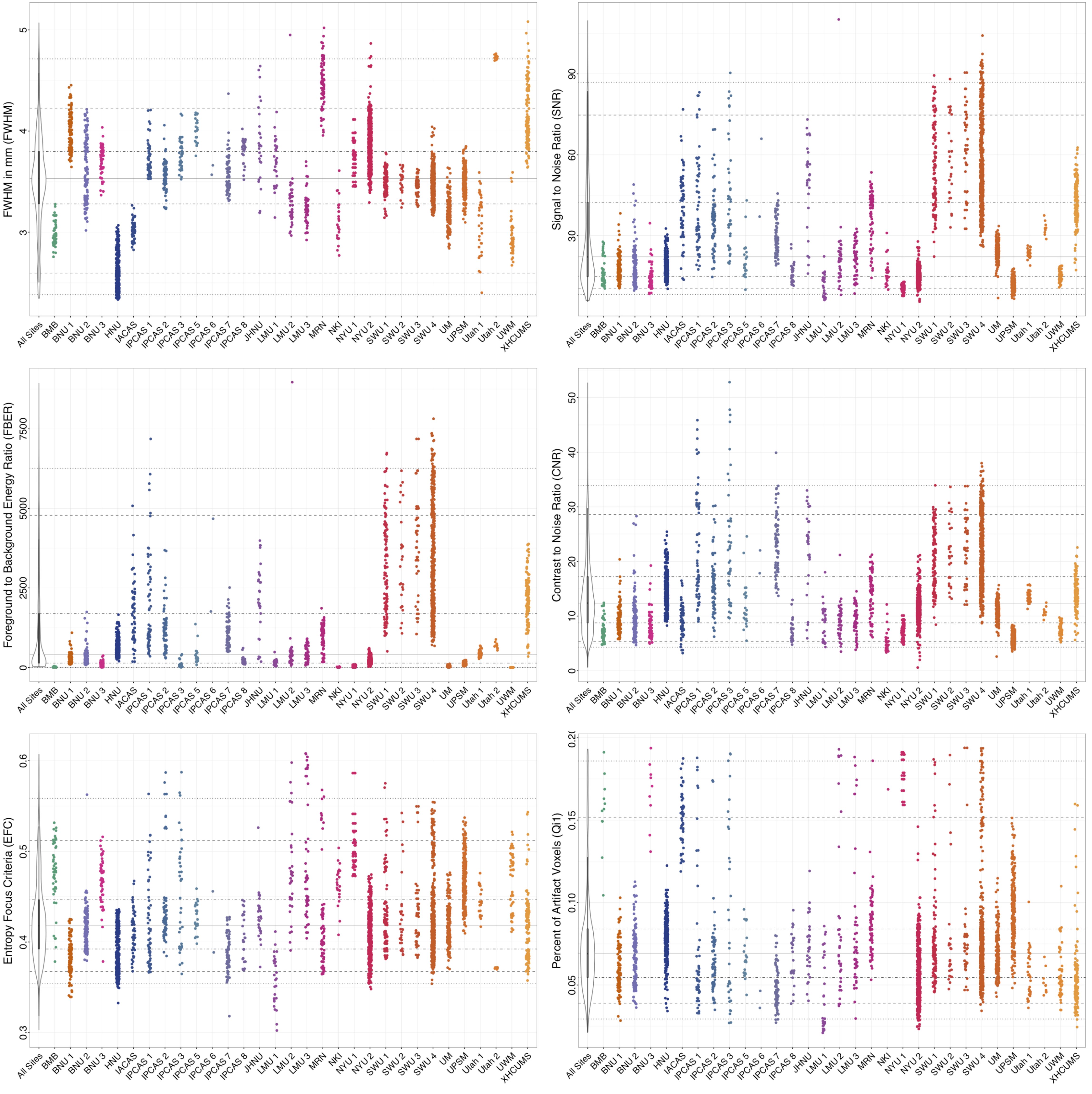
FWHM in mm (FWHM)
3
4
5
Signal to Noise Ratio (SNR)
30
60
90
Foreground to Background Energy Ratio (FBER)
0
2500
5000
7500
Contrast to Noise Ratio (CNR)
0
10
20
30
40
50
Entropy Focus Criteria (EFC)
0.3
0.4
0.5
0.6
Percent of Artifact Voxels (Qi1)
0.05
0.10
0.15
0.20
All Sites
BMB
BNU 1
BNU 2
BNU 3
HNU
IACAS
IPCAS 1
IPCAS 2
IPCAS 3
IPCAS 5
IPCAS 6
IPCAS 7
IPCAS 8
JHNU
LMU 1
LMU 2
LMU 3
MRN
NKI
NYU 1
NYU 2
SWU 1
SWU 2
SWU 3
SWU 4
UM
UPSM
Utah 1
Utah 2
UWM
XHCUMS

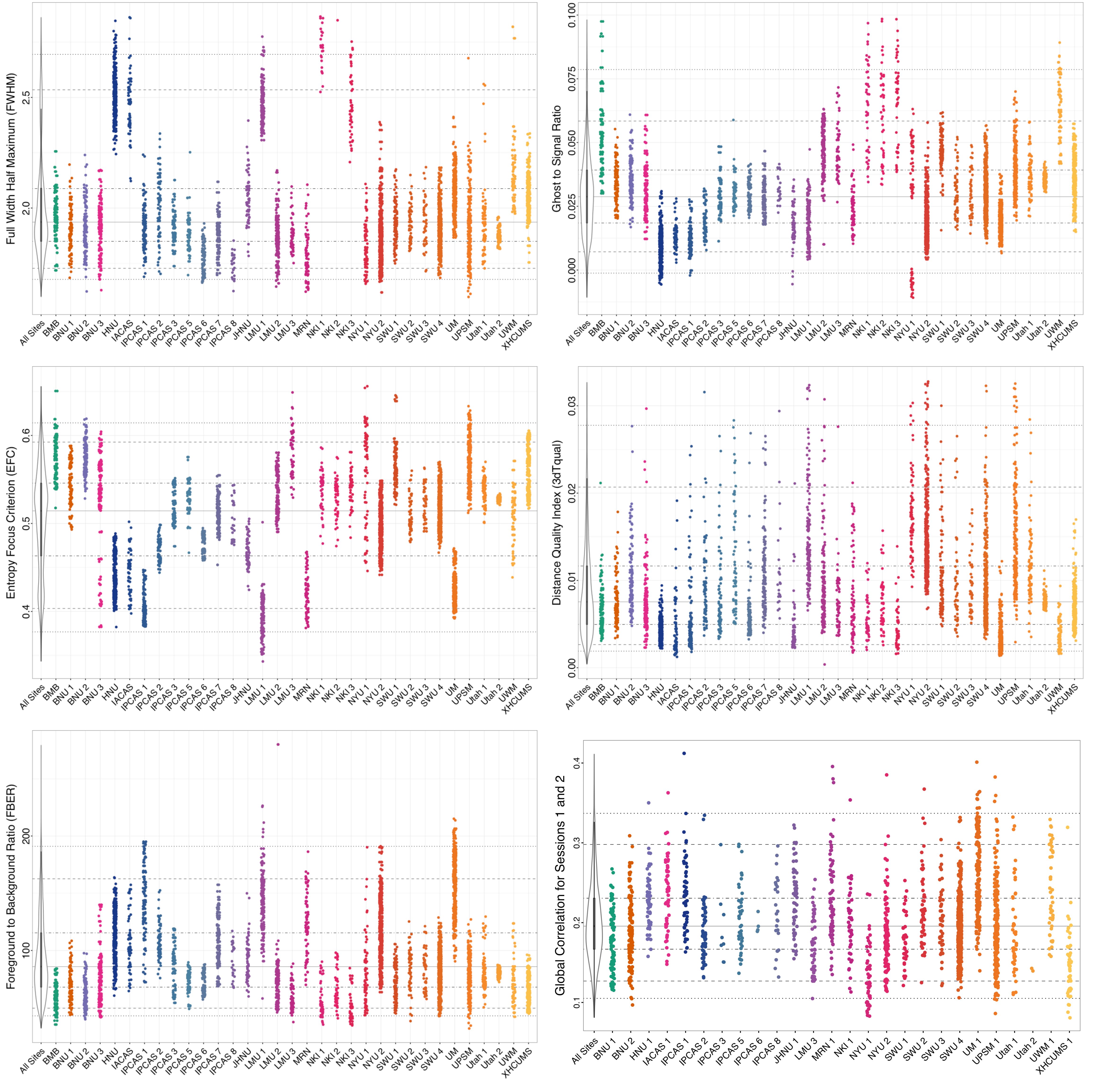

Full Width Half Maximum (FWHM)
Ghost to Signal Ratio
Entropy Focus Criterion (EFC)
Distance Quality Index (3dTqual)
Foreground to Background Ratio (FBER)
Global Correlation for Sessions 1 and 2
All Sites
BMB
BNU 1
BNU 2
BNU 3
HNU
IACAS
IPCAS 1
IPCAS 2
IPCAS 3
IPCAS 5
IPCAS 6
IPCAS 7
IPCAS 8
JHNU
LMU 1
LMU 2
LMU 3
MRN
NKI 1
NKI 2
NKI 3
NYU 1
NYU 2
SWU 1
SWU 2
SWU 3
SWU 4
UM
UPSM
Utah 1
Utah 2
UWM
XHCUMS
HNU 1
IACAS 1
IPCAS 1
JHNU 1
MRN 1
UM 1
UPSM 1
UWM 1
XHCUMS 1

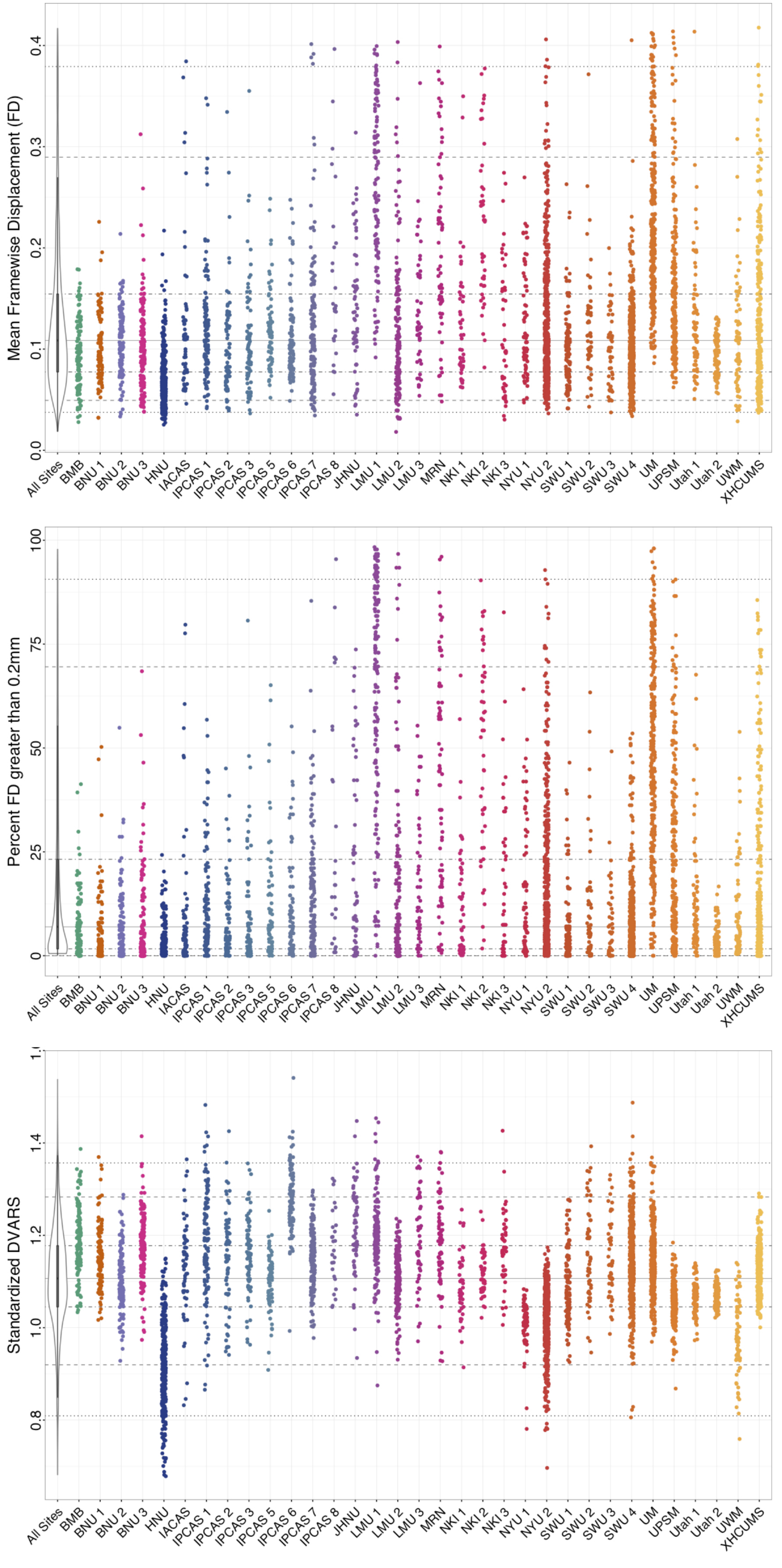

Mean Framewise Displacement (FD)
0.0
0.1
0.2
0.3
0.4
Percent FD greater than 0.2mm
0
25
50
75
100
Standardized DVARS
0.8
1.0
1.2
1.4
All Sites
BMB
BNU 1
BNU 2
BNU 3
HNU
IACAS
IPCAS 1
IPCAS 2
IPCAS 3
IPCAS 5
IPCAS 6
IPCAS 7
IPCAS 8
JHNU
LMU 1
LMU 2
LMU 3
MRN
NKI 1
NKI 2
NKI 3
NYU 1
NYU 2
SWU 1
SWU 2
SWU 3
SWU 4
UM
UPSM
Utah 1
Utah 2
UWM
XHCUMS

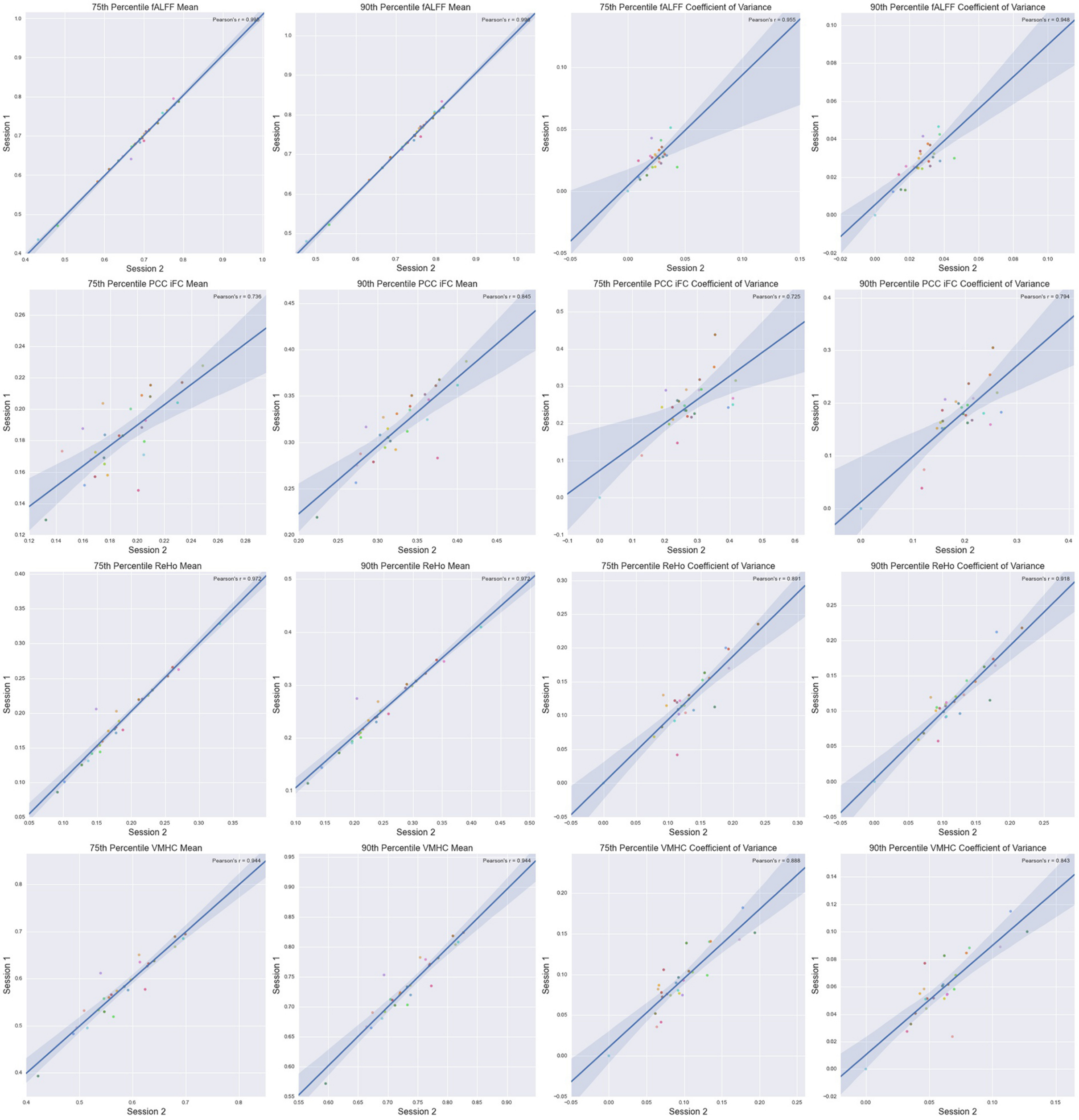

75th Percentile fALFF Mean
Pearson's r = 0.995
90th Percentile fALFF Mean
Pearson's r = 0.996
75th Percentile fALFF Coefficient of Variance
Pearson's r = 0.955
90th Percentile fALFF Coefficient of Variance
Pearson's r = 0.948
75th Percentile PCC iFC Mean
Pearson's r = 0.736
90th Percentile PCC iFC Mean
Pearson's r = 0.845
75th Percentile PCC iFC Coefficient of Variance
Pearson's r = 0.725
90th Percentile PCC iFC Coefficient of Variance
Pearson's r = 0.794
75th Percentile ReHo Mean
Pearson's r = 0.972
90th Percentile ReHo Mean
Pearson's r = 0.972
75th Percentile ReHo Coefficient of Variance
Pearson's r = 0.891
90th Percentile ReHo Coefficient of Variance
Pearson's r = 0.918
75th Percentile VMHC Mean
Pearson's r = 0.944
90th Percentile VMHC Mean
Pearson's r = 0.944
75th Percentile VMHC Coefficient of Variance
Pearson's r = 0.888
90th Percentile VMHC Coefficient of Variance
Pearson's r = 0.843
Session 1
Session 2
